# Prevalence, perinatal risk factors and clinical outcomes of respiratory *Ureaplasma* species colonization in hospitalized preterm infants

**DOI:** 10.3389/fped.2026.1773281

**Published:** 2026-02-20

**Authors:** Xiaofeng Yang, Simin Mu, Xiaolong Du, Lixiang Zhang, Xin Ding

**Affiliations:** Department of Neonatology, Children’s Hospital of Soochow University, Suzhou, Jiangsu, China

**Keywords:** clinical outcomes, preterm infants, prevalence, risk factors, *Ureaplasma* species

## Abstract

**Objective:**

To investigate the prevalence, perinatal risk factors, and clinical outcomes associated with *Ureaplasma* species (*Ureaplasma* spp.) colonization in hospitalized preterm infants.

**Methods:**

This retrospective study included preterm infants (<37 weeks’ gestation) admitted to the Neonatology Department of the Children's Hospital of Soochow University, China, between December 2023 and June 2025. Infants transferred within 72 h of birth and tested for *Ureaplasma* spp. in nasopharyngeal aspirates within 72 h were eligible. Infants with delayed testing, incomplete clinical data, or early death or discharge were excluded. Nasopharyngeal aspirates samples were analyzed for *Ureaplasma* spp. DNA by polymerase chain reaction. Demographic, perinatal, laboratory, and clinical outcome data were collected. Comparisons between *Ureaplasma* spp.-positive and *Ureaplasma* spp.-negative groups were performed, and multivariate logistic regression was used to evaluate the association between *Ureaplasma* spp. colonization and major morbidities.

**Results:**

Among 368 eligible preterm infants, 58 (15.8%) were *Ureaplasma* spp.-positive. The colonization rate increased progressively with decreasing gestational age (GA), reaching 31.8% among infants <28 weeks, and was highest among those with a birth weight of 1,000–1,499 g (20.4%). *Ureaplasma* spp.-positive infants had a significantly lower GA (*P* < 0.05). Vaginal delivery and prolonged rupture of membranes (PROM) were more common in the *Ureaplasma* spp.-positive group (both *P* < 0.001), whereas gestational hypertension was more frequent in the negative group (*P* = 0.008). The positive group had higher white blood cell counts and a greater frequency of elevated C-reactive protein (CRP) levels (*P* < 0.05). Clinically, *Ureaplasma* spp. colonization was associated with more frequent and prolonged oxygen supplementation and higher incidences of bronchopulmonary dysplasia (BPD), necrotizing enterocolitis (NEC), sepsis, and retinopathy of prematurity (ROP) (all *P* < 0.05). After adjusting for confounders, *Ureaplasma* spp. colonization remained independently associated with BPD, NEC, and ROP (*P* < 0.05), but not with sepsis.

**Conclusions:**

*Ureaplasma* spp. colonization is common in hospitalized preterm infants, particularly among those of lower gestational age. Vaginal delivery and PROM are significant perinatal risk factors. *Ureaplasma* spp. colonization is associated with heightened inflammatory responses and independently contributes to major morbidities, including BPD, NEC, and ROP, but not with sepsis after adjustment for confounders.

## Introduction

*Ureaplasma* species (*Ureaplasma* spp.) comprise *Ureaplasma parvum* (serovars 1, 3, 6, 14) and *Ureaplasma urealyticum* (serovars 2, 4, 5, 7–13). They are among the smallest prokaryotic microorganisms, intermediate in size between bacteria and viruses, and belongs to the family *Mycoplasmataceae*. As opportunistic pathogens, *Ureaplasma* spp. frequently cause urogenital tract disorders in both men and women, with particularly significant impacts on women of reproductive age. It has been reported that the detection rate of *Ureaplasma* spp. in pregnant women is as high as 82% in vaginal fluid (1). A meta-analysis reported that maternal *Ureaplasma* spp. colonization is associated with preterm birth, low birth weight (LBW), premature rupture of membranes (PROM), spontaneous abortion (SA) and/or perinatal or neonatal death (PND) (2). Moreover, increasing evidence suggests that *Ureaplasma* spp. infection during pregnancy can promote the expression of inflammatory cytokines, exacerbate inflammatory responses, interfere with inflammation clearance, and is also associated with adverse pregnancy outcomes such as premature birth and low birth weight (3).

*Ureaplasma* spp. can also be vertically transmitted to the fetus via the placenta or during passage through the birth canal, putting premature infants at a higher risk of *Ureaplasma* spp. infection. Although several studies have reported a close association between *Ureaplasma* spp. infection and neonatal diseases such as bronchopulmonary dysplasia (BPD) (4, 5), retinopathy of prematurity (ROP) (6), and sepsis, other investigations failed to demonstrate consistent associations (7).

Since the pathogenic effect of *Ureaplasma* spp. on neonatal outcomes is controversial, this study retrospectively analyzed the epidemiological characteristics and clinical outcomes of preterm infants with and without *Ureaplasma* spp. colonization in the respiratory tract, aiming to illustrate the potential clinical significance of *Ureaplasma* spp. colonization.

## Methods and materials

### Study design and participants

This retrospective study included preterm infants hospitalized in the Neonatology department of the Children's Hospital of Soochow University, China, between December 2023 and June 2025. *Ureaplasma* spp. respiratory screening was routinely performed for all preterm infants (<37 weeks' gestation) upon admission to our department, as part of standard clinical practice. Inclusion criteria were the following: (1) infants with a gestational age <37 weeks; (2) infants who were transferred to our department within 72 h after delivery; (3) infants who had been tested for *Ureaplasma* spp. in the nasal pharyngeal aspirate within 72 h of birth. Exclusion criteria were the following: (1) premature infants with *Ureaplasma* spp. detection after 72 h of birth; (2) infants with incomplete clinical data; (3) infants who died or were discharged at the parents' own request within 28 days after birth or before 36 weeks of corrected age. The neonates were divided into *Ureaplasma* spp.-positive (colonized) group and *Ureaplasma* spp.-negative based on polymerase chain reaction (PCR) results.

### Clinical sample collection and laboratory testing

Nasopharyngeal aspirate samples were collected from all preterm infants within 72 h of admission. A suction catheter was gently inserted 7–9 cm into the lower part of the pharynx for specimen collection. Respiratory specimens were tested for *Ureaplasma* spp. using a commercially available real-time polymerase chain reaction (PCR) fluorescence probe assay (*Ureaplasma* Species Nucleic Acid Detection Kit; Sansure Biotech Inc., China), according to the manufacturer's instructions. The assay is designed for qualitative detection at the genus level and does not differentiate between *Ureaplasma parvum* and *Ureaplasma urealyticum*. Bacterial load was not quantified, and results were reported as positive or negative.

### Data collection and definitions

All data during the study period were collected from the medical records: (1) perinatal status: gestational age (GA), birth weight (BW), small for gestational age (SGA), sex and mode of delivery, and maternal pregnancy status, including prolonged rupture of membranes (PROM), gestational diabetes mellitus and gestational hypertension; (2) hospitalization period: cumulative length of oxygen use and mechanical ventilation, hemogram within 3 d after birth; (3) preterm complications: presence of BPD, necrotizing enterocolitis (NEC), brain injury, ROP and other diseases.

SGA was defined as BW below the 10th percentile for GA and gender. Respiratory distress syndrome (RDS) diagnostic criteria referred to the clinical features, radiographic findings and oxygen requirement. The diagnostic criteria for sepsis in newborns were based on the 2024 Chinese expert consensus (8). BPD and its severity grades were diagnosed according to the 2001 National Institute of Child Health and Human Development (NICHD) criteria (9). “Hemodynamically significant” PDA (hsPDA) was defined by neonatal echocardiography and clinically relevant characteristics (10). NEC was defined as Bell's stage ≥II based on clinical and radiological signs (11). Infants with stage I (suspected NEC) were excluded from the analysis. ROP was defined by ophthalmologic screening according to current screening guidelines in China. White matter injury (WMI) and intracranial hemorrhage (ICH) were diagnosed by clinical examination and imaging such as magnetic resonance imaging (MRI). An abnormal C-reactive protein (CRP) level was defined as a concentration exceeding 10 mg/L (12).

### Statistical analysis

The median (interquartile spacing) [M (P25, P75)] was used for measurement data of skewness distribution, and non-parametric test was used for comparison between groups. Chi-square (*χ*^2^) test or Fisher's exact test was used for categorical data. Odds ratios (ORs) were assessed with 95% confidence intervals (CIs). Multivariate logistic regression analysis was performed to identify independent associations between *Ureaplasma* spp. colonization and neonatal outcomes. Statistical analysis was performed using SPSS software (version 22.0, IBM Corp., Armonk, NY, USA). A two-tailed *P* value <0.05 was considered statistically significant.

### Ethic approval

This study was approved by the Medial Ethics Committees of the Children's Hospital of Soochow University (2024CS098).

## Results

### Prevalence and demographic characteristics

**Figure 1:**
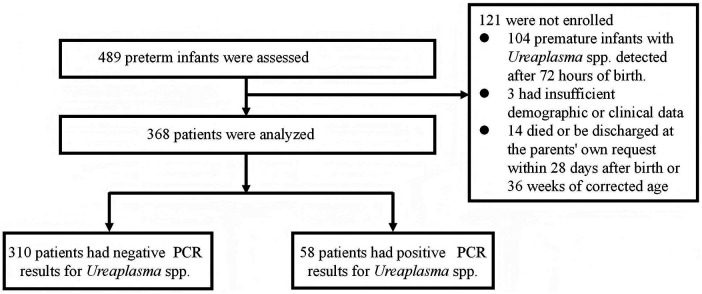
Flow of study participants.

Among 489 preterm infants admitted during the study period, 104 were excluded due to *Ureaplasma* spp. testing more than 72 h after birth, 3 due to incomplete data, and 14 due to early death or discharge. A total of 368 infants met the inclusion criteria (Figure 1).

**Figure 2:**
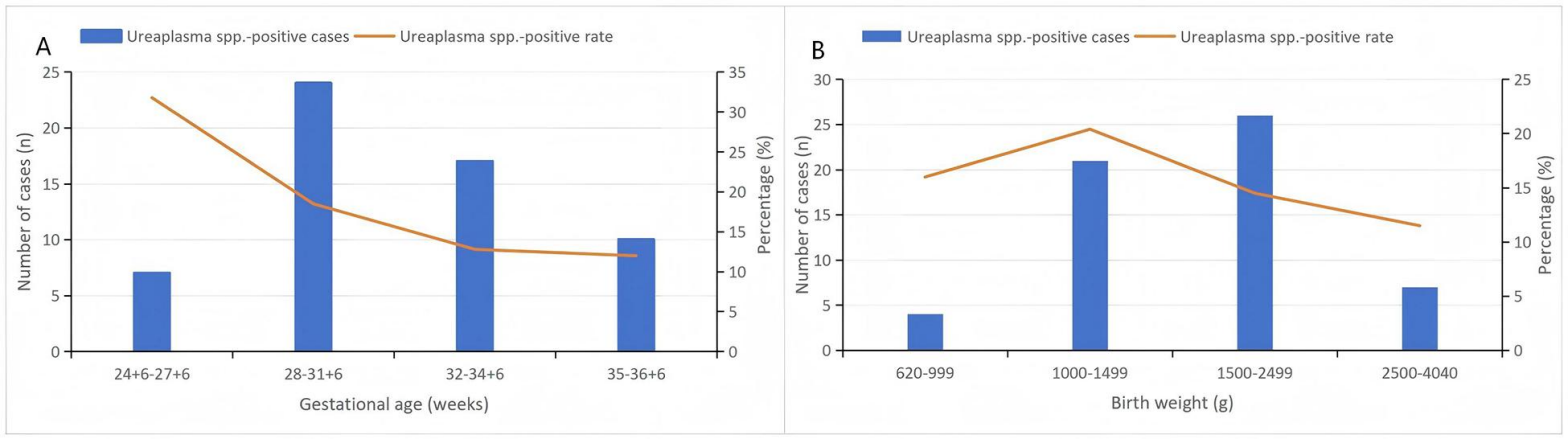
Distribution of *Ureaplasma* spp. colonization by gestational age and birth weight. **(A)** Percentage and number of infants with *Ureaplasma* spp. colonization stratified by gestational age groups. **(B)** Percentage and number of infants with *Ureaplasma* spp. colonization stratified by birth weight categories.

The prevalence of respiratory tract *Ureaplasma* spp. colonization was 15.8% (58/368). The mean GA of the cohort was 32.4 weeks (range: 24 weeks +6 days to 36 weeks +6 days), and the mean BW was 1,832.7 g (range: 620–4,040 g). The colonization rate increased progressively with decreasing gestational age (GA), reaching 31.8% among infants < 28 weeks. The highest detection rate by birth weight (BW) category was observed in the 1,000–1,499 g group (20.4%) (Figure 2).

### Perinatal and laboratory characteristics associated with *Ureaplasma* spp. colonization

Table 1 depicts the perinatal and laboratory characteristics of preterm neonates with and without *Ureaplasma* spp. colonization. GA was significantly lower in the *Ureaplasma* spp.-positive group (*P* < 0.05), while BW, SGA, and sex distribution did not differ significantly (*P* > 0.05).

**Table 1:** Comparison of perinatal and laboratory characteristics between preterm neonates with and without *Ureaplasma* spp. colonization.

Variables	*Ureaplasma* spp. negative (*n* = 310)	*Ureaplasma* spp. positive (*n* = 58)	*P* value
Male, *n* (%)	175 (56.5)	35 (60.3)	0.582
GA (weeks), median (IQR)	32.9 (30.7, 34.7)	31.6 (29.1, 34.0)	**0**.**008**
BW (g), median (IQR)	1,795.0 (1,317.0, 2,300.0)	1,635.0 (1,250.0, 2,092.5)	0.090
SGA, *n* (%)	29 (9.4)	1 (1.7)	0.064
PROM, *n* (%)	99 (31.9)	33 (56.9)	**<0**.**001**
Vaginal delivery, *n* (%)	61 (19.7)	38 (65.5)	**<0**.**001**
Gestational hypertension	96 (31.0)	8 (13.8)	**0**.**008**
Gestational diabetes mellitus	57 (18.4)	12 (20.7)	0.680
1-min Apgar score, median (IQR)	9 (8, 10)	9 (8, 10)	0.244
5-min Apgar score, median (IQR)	10 (9, 10)	10 (9, 10)	0.298
White blood cell count (×10^9^/L), median (IQR)	9.5 (6.7, 13.9)	12.2 (8.6, 20.3)	**<0**.**001**
Platelet count(×10^9^/L), median (IQR)	236.0 (186.5, 286.0)	244.0 (191.8, 315.3)	0.284
Abnormal CRP level, *n* (%)	14 (4.5)	7 (10.3)	**0**.**023**

GA, gestational age; BW, birth weight; PROM, prolonged rupture of membranes; IQR, interquartile range.

Bold values indicate statistical significance (*P* < 0.05).

Among perinatal factors, vaginal delivery and PROM were significantly more common in the *Ureaplasma* spp.-positive group than in the *Ureaplasma* spp.-negative group (both *P* < 0.001), whereas gestational hypertension was more frequent in the negative group (*P* = 0.008). No significant differences were observed in the incidence of gestational diabetes mellitus, or in 1-min Apgar score and 5-min Apgar score.

Regarding laboratory findings, the levels of white blood cells [12.2 (8.6, 20.3)10 × ^9^/L], and the frequency of elevated CRP levels in *Ureaplasma* spp.-positive group were higher than the negative group. The differences were all statistically significant (*P* < 0.05). However, platelet count did not differ between the two groups (*P* > 0.05).

### Outcomes of preterm infants with and without *Ureaplasma* spp. colonization

Compared with *Ureaplasma* spp.-negative infants, *Ureaplasma* spp.-positive infants required oxygen supplementation more frequently and had a longer duration of oxygen therapy (both *P* < 0.05). However, there were no significant differences in the incidence of invasive mechanical ventilation, or duration of invasive mechanical ventilation (*P* > 0.05).

The incidences of BPD, NEC, and ROP were significantly higher in the *Ureaplasma* spp.-positive group than in the negative group (all *P* < 0.05). Both mild and moderate-to-severe BPD occurred more frequently among *Ureaplasma* spp.-positive infants. In addition, a higher proportion of stage III NEC and higher-stage ROP (stage >3) was observed in the *Ureaplasma* spp.-positive group. No significant differences were found in the incidence of RDS, hsPDA, WMI, ICH, or detection of respiratory potentially pathogenic bacteria (all *P* > 0.05) (Table 2).

**Table 2:** Outcomes of preterm infants with and without *Ureaplasma* spp. colonization.

Variables	*Ureaplasma* spp. negative (*n* = 310)	*Ureaplasma* spp. positive (*n* = 58)	*P* value
Any form of oxygen supplementation, *n* (%)	272 (87.7)	57 (98.3)	**0**.**017**
Duration of oxygen administration (days), median (IQR)	18.0 (8.0, 39.0)	25.0 (10, 56)	**0**.**015**
Invasive mechanical ventilation, *n* (%)	105 (33.9)	23 (39.7)	0.396
Duration of invasive mechanical ventilation (days), median (IQR)	0 (0, 4)	0 (0, 4.5)	0.424
RDS, *n* (%)	149 (48.1)	25 (43.1)	0.487
hsPDA, *n* (%)	26 (8.4)	7 (12.1)	0.368
BPD, *n* (%)	39 (12.6)	15 (25.9)	**0**.**009**
Mild BPD, *n* (%)	18 (5.8)	5 (8.6)	**—**
Moderate—severe BPD, *n* (%)	21 (6.8)	10 (17.2)	**—**
NEC, *n* (%)	18 (5.8)	8 (13.8)	**0**.**029**
Stage II, *n* (%)	13 (4.2)	4 (6.9)	**—**
Stage III, *n* (%)	5 (1.6)	4 (6.9)	**—**
Sepsis, *n* (%)	27 (8.7)	10 (17.2)	**0**.**047**
ROP, *n* (%)	21 (6.8)	12 (20.7)	**0**.**001**
Stage 1–2, *n* (%)	19 (6.1)	8 (13.8)	**—**
Stage ≥ 3, *n* (%)	2 (0.6)	4 (6.9)	**—**
WMI, *n* (%)	5 (1.6)	2 (3.4)	0.348
ICH, *n* (%)	72 (23.3)	16 (27.6)	0.483
Respiratory potentially pathogenic bacteria detected, *n* (%)	13 (4.2)	4 (6.9)	0.368

IQR, interquartile range;RDS, respiratory distress syndrome; hsPDA, hemodynamically significant patent ductus arteriosus; BPD, bronchopulmonary dysplasia; NEC, necrotizing enterocolitis; ROP, retinopathy of prematurity; WMI, white matter damage; ICH, intracranial hemorrhage.

NEC was defined as Bell's stage ≥II.

Bold values indicate statistical significance (*P* < 0.05).

Table 3 shows that, after adjusting for GA, BW, sex, mode of delivery, gestational hypertension, gestational diabetes mellitus, PROM, and hsPDA, *Ureaplasma* spp. colonization remained independently associated with the development of BPD, NEC and ROP (all *P* < 0.05). However, no statistically significant association was observed between *Ureaplasma* spp. colonization and sepsis (*P* > 0.05).

**Table 3:** Multivariable logistic regression of *Ureaplasma* spp. colonization and clinical outcomes in preterm infants.

Variables	OR	95% CI	*P* value
BPD	3.152	1.026–9.681	0.045
NEC	4.274	1.267–14.411	0.019
ROP	3.943	1.216–12.788	0.022
Sepsis	2.591	0.991–6.771	0.052

BPD, bronchopulmonary dysplasia; NEC, necrotizing enterocolitis; ROP, retinopathy of prematurity; OR, odds ratio; CI, confidence interval.

NEC was defined as Bell's stage ≥II. All adjusted models included the following covariates: adjusting for gestational age, birth weight, sex, mode of delivery, gestational hypertension, gestational diabetes mellitus, prolonged rupture of membranes, and hemodynamically significant patent ductus arteriosus.

## Discussion

In this retrospective study, we analyzed the overall detection rate of *Ureaplasma* spp. among preterm neonates, with attention to GA and BW. The detection rate of *Ureaplasma* spp. among hospitalized preterm neonates in our cohort was 16.3%, while previous studies revealed different positive rates of *Ureaplasma* spp. in preterm neonates (13–15). As a referral center, we are more likely to receive clinically unstable or high-risk preterm infants, which may partly explain the high detection rate of *Ureaplasma* spp. in our study.

In our study, the prevalence of *Ureaplasma* spp. colonization in the respiratory tract increased progressively with decreasing GA, reaching 31.8% among infants born before 28 weeks' gestation. In contrast, the highest detection rate by BW category was observed in the 1,000–1,499 g group. This pattern suggests that *Ureaplasma* spp. colonization is more closely related to the degree of prematurity than to BW. It has been reported that the lower GA, the higher the vertical transmission rate, aligning with our findings (16). Furthermore, the immature epithelial barrier and underdeveloped immune system of extremely preterm infants may facilitate bacterial adherence and persistence in the respiratory tract. The slightly lower detection rate observed in infants weighing <1,000 g compared with those in the 1,000–1,499 g group may be attributable to the small sample size of this subgroup, the early use of empirical antibiotics or a higher likelihood of early death or discharge at the parents' own request, which may have reduced the bacterial load below the detection threshold.

Consistent with previous studies (5, 17, 18), we observed a higher incidence of PROM (56.9%) and a higher percentage of vaginal deliveries (65.5%) among mothers of *Ureaplasma* spp.-positive infants compared with those of *Ureaplasma* spp.-negative infants, supporting the notion that vertical transmission is the dominant route of acquisition (19). The lower incidence of gestational hypertension among mothers of *Ureaplasma* spp.-positive neonates in our study may reflect underlying differences in the mechanisms leading to preterm birth rather than a protective effect of gestational hypertension itself. In addition, *Ureaplasma* spp.-positive neonates in our study exhibited higher white blood cell counts and a greater frequency of elevated CRP, consistent with earlier investigations (5, 15, 18). Colonization of the respiratory tract by *Ureaplasma* has been associated with both acute and persistent inflammatory activation in preterm infants, reflected by leukocytosis and elevated high-sensitivity CRP. The inflammatory response triggered by *Ureaplasma* spp. infection likely contributes to the increased circulating inflammatory markers observed in colonized neonates (20).

In our study, the incidence of BPD was higher in the *Ureaplasma* spp.-positive group, with both mild and moderate-to-severe BPD occurring more frequently among *Ureaplasma* spp.-positive infants. These findings are consistent with numerous prior studies that have shown a link between *Ureaplasma* spp. colonization and BPD (4, 5, 18, 21). Although some studies have suggested that this association may be confounded by GA, BW, or perinatal interventions, the relationship in our cohort remained significant after adjusting for these factors, supporting the clinical relevance of *Ureaplasma* spp. colonization as an independent risk factor for BPD. The proposed mechanisms may involve *Ureaplasma* spp.-induced persistent pulmonary inflammation, interstitial pulmonary edema, impaired alveolar development, and exacerbation of the deleterious effects of oxygen on lung development, ultimately contributing to the development of BPD (22, 23). In line with this, *Ureaplasma* spp.-positive preterm infants in our study more frequently required oxygen supplementation and had longer durations of oxygen therapy (*P* < 0.05), suggesting that *Ureaplasm*a colonization may contribute to greater respiratory injury in preterm neonates. The role of macrolide therapy in *Ureaplasma* respiratory colonization remains controversial. The AZTEC trial reported no significant reduction in BPD incidence with macrolide treatment (24), suggesting that *Ureaplasma* eradication alone may be insufficient to prevent chronic lung disease. In our study, macrolides were administered to only a limited number of infants, and treatment effects were not evaluated.

It has been reported that the incidence of NEC is higher among *Ureaplasma* spp.-positive infants compared with *Ureaplasma* spp.-negative infants (25). Our findings similarly demonstrated an association between *Ureaplasma* spp. colonization and the incidence of NEC, suggesting that *Ureaplasma* spp. colonization may be linked to a systemic inflammatory response rather than being limited to the respiratory tract. *Ureaplasma* spp. exposure has been shown to be associated with fetal intestinal inflammation and with alterations in enterocyte proliferation, differentiation, and maturation (26). Moreover, *Ureaplasma* spp.-driven chorioamnionitis has been reported to be associated with thickening of the ileal mucus layer and increased mucus secretion by goblet cells (27). The overlaps between these findings and pathological characteristics observed in NEC support the biological plausibility of an association between *Ureaplasma* spp. exposure and intestinal injury.

Previous studies have reported inconsistent findings regarding the association between *Ureaplasma* and ROP (13, 17, 18). In our study, *Ureaplasma* spp colonization was found to be associated with ROP, which is consistent with the results reported by Ma et al. (17) and Sun et al. (13). *Ureaplasma* spp. exposure has been shown to trigger fetal inflammatory response syndrome (28) and alter microvascular development (29), both of which have been implicated in the pathogenesis of abnormal retinal vascularization. Furthermore, *Ureaplasma* spp. colonization has been associated with lower birth weight, which may further increase susceptibility to the development of ROP.

This single-center study with a modest sample size may have several limitations. *Ureaplasma* spp. load was not quantified, and the PCR assay detected organisms only at the genus level, precluding dose-response and species-specific analyses. Detection was based on a single nasopharyngeal aspirate, which may have underestimated colonization and could not distinguish transient from persistent colonization, colonization from infection, or lower respiratory tract involvement. Exclusion of infants who died early may have removed death as a competing outcome. In addition, maternal chorioamnionitis diagnoses and placental pathology were unavailable in this tertiary pediatric cohort, precluding assessment of fetal inflammatory response.

## Conclusion

In conclusion, the rate of *Ureaplasma* spp. colonization increased progressively with decreasing gestational age among preterm infants. Compared with non-colonized infants, those with *Ureaplasma* spp. colonization had a significantly lower GA, and were more likely to be delivered vaginally and to have a history of PROM. In addition, *Ureaplasma* spp.-positive infants exhibited higher WBC counts and a greater frequency of elevated CRP levels. Clinically, *Ureaplasma* spp. colonization was associated with increased oxygen requirements and a prolonged duration of oxygen therapy. Importantly, our findings suggest that *Ureaplasma* spp. colonization is independently associated with multiple major morbidities, including BPD, NEC, and ROP. These results underscore the potential clinical importance of early identification and targeted management of *Ureaplasma* spp. colonization in preterm infants.

## Data Availability

The data analyzed in this study is subject to the following licenses/restrictions: The data that support the findings of this study are available from our hospital database, but restrictions apply to the availability of these data, which were used under license for the current study, and so are not publicly available. Requests to access these datasets should be directed to Xin Ding, 68820717@qq.com.
